# Frequent p53 mutation in brain (fetal)-type glycogen phosphorylase positive foci adjacent to human ‘de novo’olorectal carcinomas

**DOI:** 10.1054/bjoc.2001.1824

**Published:** 2001-06

**Authors:** S Shimada, K Shiomori, S Tashima, J Tsuruta, M Ogawa

**Affiliations:** Department of Surgery II, andDepartment of Surgical Pathology, Kumamoto University School of Medicine, 1-1-1 Honjo, Kumamoto 860-8556, Japan

**Keywords:** colorectal carcinoma, ‘de novo’, carcinoma, glycogen phosphorylase, p53, K-ras, transitional mucosa

## Abstract

‘de novo’ carcinogenesis has been advocated besides ‘adenoma carcinoma sequence’ as another dominant pathway leading to colorectal carcinoma. Our recent study has demonstrated that the distribution of brain (fetal)-type glycogen phosphorylase (BGP) positive foci (BGP foci) has a close relationship with the location of ‘de novo’ carcinoma. The aims of the present study are to investigate genetic alteration in the BGP foci and to characterize them in the ‘de novo’ carcinogenesis. 17 colorectal carcinomas without any adenoma component expressing both immunoreactive p53 and BGP protein were selected from 96 resected specimens from our previous study. Further investigations to examine the proliferating cell nuclear antigen (PCNA)-labelling index, and the p53 and the codon 12 of K-ras mutation using the polymerase chain reaction-single strand conformation polymorphism were performed in the BGP foci, BGP negative mucosa and carcinoma. The BGP foci were observed sporadically in the transitional mucosa adjacent to the carcinoma in all cases. The PCNA labelling index in the BGP foci was significantly higher than that in the BGP negative mucosa (*P*< 0.001). p53 mutations were observed in 8 carcinomas, but no K-ras mutation was detected. Interestingly, although none of the overexpressions of p53 protein was detected immunohistochemically in the BGP positive foci, the *p53* gene frequently (41.2% of the BGP foci tested) mutated in spite of no K-ras mutation. The present study demonstrates potentially premalignant foci in the colorectal transitional mucosa with frequent *p53* gene mutation. It is suggested that BGP foci are promising candidates for the further investigation of ‘de novo’ colorectal carcinogenesis. © 2001Cancer Research Campaign http://www.bjcancer.com


*British Journal of Cancer* (2001) **84**(11), 1497–1504
© 2001 Cancer Research Campaign
doi: 10.1054/ bjoc.2001.1824, available online at http://www.idealibrary.com on IDEAL®

### Frequent p53 mutation in brain (fetal)-type glycogen phosphorylase positive foci adjacent to human ‘de novo’ colorectal carcinomas

**S Shimada[1], K Shiomori[1], S Tashima[1], J Tsuruta[2] and M Ogawa[1]**

[1]Department of Surgery II, and [2]Department of Surgical Pathology, Kumamoto University School of Medicine, 1-1-1 Honjo, Kumamoto 860-8556, Japan

**Summary** ‘de novo’ carcinogenesis has been advocated besides ‘adenoma carcinoma sequence’ as another dominant pathway leading to colorectal carcinoma. Our recent study has demonstrated that the distribution of brain (fetal)-type glycogen phosphorylase (BGP) positive foci (BGP foci) has a close relationship with the location of ‘de novo’ carcinoma. The aims of the present study are to investigate genetic alteration in the BGP foci and to characterize them in the ‘de novo’ carcinogenesis. 17 colorectal carcinomas without any adenoma component expressing both immunoreactive p53 and BGP protein were selected from 96 resected specimens from our previous study. Further investigations to examine the proliferating cell nuclear antigen (PCNA)-labelling index, and the p53 and the codon 12 of K-ras mutation using the polymerase chain reaction-single strand conformation polymorphism were performed in the BGP foci, BGP negative mucosa and carcinoma. The BGP foci were observed sporadically in the transitional mucosa adjacent to the carcinoma in all cases. The PCNA labelling index in the BGP foci was significantly higher than that in the BGP negative mucosa ($P < 0.001$). p53 mutations were observed in 8 carcinomas, but no K-ras mutation was detected. Interestingly, although none of the overexpressions of p53 protein was detected immunohistochemically in the BGP positive foci, the *p53* gene frequently (41.2% of the BGP foci tested) mutated in spite of no K-ras mutation. The present study demonstrates potentially premalignant foci in the colorectal transitional mucosa with frequent *p53* gene mutation. It is suggested that BGP foci are promising candidates for the further investigation of ‘de novo’ colorectal carcinogenesis. © 2001 Cancer Research Campaign http://www.bjcancer.com

**Keywords**: colorectal carcinoma; ‘de novo’ carcinoma; glycogen phosphorylase; p53; K-ras; transitional mucosa

The 2 pathways of ‘adenoma-carcinoma sequence’ (ACS) (Morson, 1974; Vogelstein et al, 1988; Fearon and Jones, 1992) and ‘de novo’ carcinogenesis (Shamsuddin et al, 1985; Kuramoto and Oohara, 1988; Shimoda et al, 1989; Rembacken et al, 2000) have been advocated as the representative mechanisms leading to the sporadic colorectal carcinoma. In the former, carcinoma is thought to arise from the pre-existing adenoma, and in the latter it is thought to develop directly from the non-neoplastic colorectal epithelia. Although the multistage progressive processes in the ACS have been elucidated using various oncogenes and tumour suppressor genes (Vogelstein et al, 1988; Fearon and Jones, 1992), the scenario of ‘de novo’ carcinogenesis has little genetic evidence to establish this pathway. To date, it has been suggested that p53 has a more important role in the development of ‘de novo’ carcinoma than K-ras, which frequently involves in the ACS theory (Yamagata et al, 1994; Minamoto et al, 1994; Mueller et al, 1996). However, if some mucosal change in the normal appearing colorectal mucosa can not be identified as a candidate for premalignant lesion, it may be impossible to investigate the ‘de novo’ carcinoma pathway.

Glycogen phosphorylase (GP) (EC 2.4.1.1) plays a central role in the mobilization of carbohydrate reserves in a wide variety of organs and tissues, and is one of the most carefully investigated enzymes in history (Cori and Cori, 1936; Krebs and Fisher, 1956; Davis et al, 1967; Newgard et al, 1989). Mammalian GPs are found in 3 major isoforms, i.e. muscle, liver and brain (BGP), that can be distinguished by functional and structural properties as well as by the tissues in which they are predominantly expressed (Shimada et al, 1986; Newgard et al, 1989). cDNAs encoding the 3 human GP isoforms have been cloned and sequenced (Nakano et al, 1986; Newgard et al, 1988). The physiological role of BGP is poorly understood, but it is generally thought to induce an emergency glucose supply during a stressful and ischaemic period including neoplastic state. In addition, it has been suggested that the major isoform of GP found in fetal and tumour tissues is BGP, and BGP has been demonstrated to be identical with fetal-type GP (Sato et al, 1972; Shimada et al, 1987, 1999; Gelinas et al, 1989).

We have recently demonstrated BGP expression common in the colorectal carcinomas, increasing in the ACS multisteps, and frequent in the transitional mucosa of colorectal carcinoma without any adenoma component (Tashima et al, 2000). The BGP visualized by immunohistochemistry was commonly present in colorectal carcinoma (80/96: 83.3%). The expression of this molecule during ACS showed excellent correlation with the increased dysplasia and was found prior to p53 expression, whereas no BGP expression was seen in the normal human large intestine remote from the cancer foci. Positive BGP staining in colorectal mucosa (BGP focus) both in the proliferating zone and epithelial cells was observed mainly around pure carcinomas without any adenoma component. These results indicate that the BGP focus may contribute to the carcinogenesis of the ‘de novo’ carcinoma. Adjacent mucosa around existing carcinoma, the so-called ‘transitional

*Received 25 September 2000*
*Revised 7 December 2000*
*Accepted 25 January 2001*

*Correspondence to:* M Ogawa mucosa' (Filipe and Cooke, 1974), has been advocated as a distinct entity which is indicative of premalignant changes such as high proliferative activity (Wang et al, 1992) or K-ras mutation (Zhang et al, 1998) in the crypt stem cell. These observations support the contention that a malignant field change exists in transitional mucosa adjacent to a colorectal carcinoma.

In the present study, the BGP foci are further investigated using proliferating analysis and genetic alterations, and we discuss these BGP foci as a potentially precancerous lesion of 'de novo' carcinoma.

### MATERIALS AND METHODS

### Tissues

96 human specimens were surgically resected from patients with colorectal carcinoma. Tissues were fixed in 10% buffered formalin for 4 days, sliced approximately 5 cm in length, embedded in paraffin, and cut into serial 5 sections for histological and immunohistochemical examinations. The first section in those was stained with haematoxylin and eosin, and examined under a light microscope.

To investigate the 'de novo' carcinogenesis, 17 colorectal carcinomas with invasion into the submucosa or superficial muscularis propria (upper one-third of muscularis propria) without any adenoma component expressing both immunoreactive p53 and BGP protein were selected from the 96 resected specimens. The mean size of the seventeen carcinoma were 2.6 ± 0.9 cm (mean ± SD) in diameter. All patients were Japanese. Age ranged from 38 to 81 (62 ± 12: mean ± SD), and the ratio of male to female was 9:8. 3 serial sections of 3 µm for PCNA, p53 and BGP staining *each*, and 5 to 8 µm were prepared for further examination of K-ras and p53 mutation.

### Antibodies

Antibody against human BGP was raised as previously reported by Ignacio et al (1990) with modification. Briefly, a synthesized 13-residue peptide (CDLQIPPPNIPRD) corresponding cystein coupled to the 12 carboxyterminal residues of BGP was chosen for immunogen, which had no significant homology with other proteins including human liver and muscle-type GP using GENE WORKS homology search. The N-terminal cysteine of the peptide was coupled with activated keyhole limpet haemocyanin (KLH) (PIERCE, Illinois, USA). Adult rabbits were immunized by 4 subcutaneous injections at one week intervals, with 100 µg of the KLH-peptide with Freund's complete or incomplete adjuvant. One week after the last injection the same amount of KLH-peptide was injected subcutaneously as a booster, and one week later blood samples were collected from the jugular vein. The immunoglobulin G (IgG) fractions of pooled antisera (approximately 100 ml) were precipitated by adding saturated $(NH_4)_2SO_4$ (50% saturation), dissolved in 100 ml of PBS and dialysed extensively in the same buffer. A part of this IgG fraction (2 ml) was then applied to a column of BGP-coupled Sepharose (1 mg of the BGP peptide was coupled with 1 ml of Hi Trap NHS-activated Sepharose) (Pharmacia Biotech, Uppsala, Sweden) at a flow rate of 0.5 ml $min^{-1}$ at room temperature. The column was washed successively with buffer A (0.5 M ethanolamine, 0.5 M NaCl, pH 8.3) and buffer B (0.1 M acetate buffer, 0.5 M NaCl, pH 4.0). Then the antibodies bound in the column were eluted with 0.1 M glycine-HCl buffer, pH 2.5, containing 1 M NaCl. Fractions containing antibodies, which were detected by measuring the absorbance at 280 mn, were collected, neutralized immediately with 1 M NaOH, and dialysed against PBS buffer.

Anti-p53 Ab (BP53.12) and anti-proliferating cell nuclear antigen (PCNA) Ab (PC-10) was purchased from ZYMED Laboratory Inc (San Francisco, CA, USA) and Medac (Hamburg, Germany), respectively.

### Immunohistochemistry

The ABC Elite kits (Vector Laboratories, Inc. Burlingame, CA, USA) for rabbit IgG (anti-BGP) and mouse IgG (anti-p53 and anti-PCNA) were used. Sections of formalin-fixed and paraffin-embedded tissue (3 µm thick) were deparaffinized and hydrated through xylene and graded ethanols. Sections were incubated with normal horse serum for 30 min following the blocking of endogenous peroxidase activity with 0.3% $H_2O_2$ in methanol for 30 min, incubated overnight at 4˚C with optimally diluted (see below) primary Ab, and subsequently incubated with biotinylated anti-rabbit and -mouse IgG Ab and avidin-biotin peroxidase complex for 30 min at room temperature. They were washed in 10 mM PBS (pH 7.2) between each incubation step. The sites of peroxidase activity were visualized by the diaminobenzidine method. Sections were counter stained with haematoxylin for microscopic examination. In p53 staining, heat antigen retrieval was used (Shi et al, 1991). Sections were immersed in 1 l of citrate buffer (2.1 g citric acid in 1 l distilled water: pH 6.0 with 2 M NaOH) and boiled 30 min before the immunohistochemical procedure. The working dilutions of the primary antibodies employed in this study were 1 µg $ml^{-1}$ of the affinity purified anti-BGP Ab, and 1:800 dilution for PC-10 (anti-PCNA Ab). BP53.12 (anti-p53 Ab) prediluted by the manufacturer was directly applied without additional dilution. As a negative control, non-immunized rabbit and mouse IgG were used instead of the primary Ab.

### PCNA labelling index

The PCNA labelling index of colorectal carcinoma was determined as the percentage of immunoreactive nuclei in 1000 cells. In colorectal mucosa, PCNA labelling index was expressed as the percentage of positive cells in a longitudinally sectioned colorectal tubule. Calculated data were expressed as mean ± SD.

### Microdissection

Based upon BGP staining of 3 µm section, each part of the carcinoma with positive BGP staining, BGP focus, and BGP negative colorectal mucosa were collected in microtubes from all of the 5 8 µm serial sections using needles (0.41 mm outer diameter). To eliminate the contamination of the carcinoma cells, the BGP negative colorectal mucosa was collected first, subsequently the BGP foci, and at last the carcinoma part using new needles. Genomic DNA was extracted from tissues by proteinase K digestion and phenol/chloroform extraction (Sambrook et al, 1989), with minor modifications.

### Primers

Oligonucleotides were synthesized as primers for PCR based on the published *p53* gene sequence for each region flanking the

 *© 2001 Cancer Research Campaign*

intron/exon 5 to 8 (Buchman et al, 1988). The designations and sequences for each primer were as follows; EX-05f: 5′-TCTGTCT-CCCTTCCTCTTCCT-3′, EX-05r: 5′-TCTCCAGCCCCAGCT-GCT-3′, EX-06f: 5′-TGATTCCTCACTGATTGCTCT-3′, EX-F05r:F-5′-TCTCCAGCCCCAGCTGCT-3′,EX-06f:5′-TGATCCT CACTGATTGCTCT-3′, EX-06r: 5′-GAGACCCCAG-TTGCCA-AACC-3′, EX-F06r: F-5′-GAGACCCCAGTTGCCAAACC-3′, EX-07f: 5′-TCTTGGGCCTGTGTTATCTC3′, EX-07: 5′-AGGGTGGCAAGTGGCTCC-3′, EX-F07r: F-5′-AGGG TGGCAAGTGGCTCC-3′, EX-08f: 5′GCTTCTCTTTTCCTATC-CTGA-3′, EX-08r: 5′-CGCTTCTTGTCCTGCTTGC-3′, and EX-F08r: F-5′-CGCTTCTTGTCCTGCTTGC-3′. The number in each designation indicates the region of the exon of the *p53* gene subjected to examination by polymerase chain reaction-single strand conformation polymorphism (PCR-SSCP) or fluorescence-based PCR-SSCP (PCR-FSSCP) analysis. 'f' and 'r' indicate forward and reverse primers respectively, for each region. The reverse primers were labeled at their 5′ ends with fluorescein derivatives by the Fluore prime method (Pharmacia Biotech, Uppsala, Sweden). 'F' indicates carboxyfluorescein linked to the 5′-nucleotide via a linker and phosphate.

We used synthesized oligonucleotides, K1203f: 5′-GTACTG-GTGGAGTATTTGATAGT-3′ and K1204r: 5′CATGAAAATG-GTCAGAGAAACC-3′, as primers for codon 12 of K-ras PCR-SSCP analysis. 'f' and 'r' indicate forward and reverse primers respectively.

### PCR-SSCP analysis

The target sequences were amplified by PCR in 50 μl of solution containing 1 μM concentrations of the primers, 100 μM concentrations of dATP, dCTP, dGTP and TTP, 100 ng of human genomic DNA, and 0.125 units of Amplitaq (Perkin-Elmer Cetus, Norwalk, CT, USA) in the buffer recommended for the enzyme. All PCR reactions were performed in a Robo-Cycler™ (STRATAGENE, La Jolla, CA, USA) over 40 cycles. Each reaction cycle included denaturation at 95˚C for 1 min, primer annealing at 56˚C for 1.5 min, and primer extension at 72˚C for 1.5 min. 10 μl of PCR products were diluted 2-fold by a buffer consisting of 20 mM EDTA, 96% formamide, 0.05% bromophenol blue, and 0.05% xylene cyanol and heated at 95˚C for 5 min, and then placed on ice for 5 min. Then 10 μl of this solution was applied to each lane of a 7.5% neutral small polyacrylamide gel (8 cm × 9 cm). Electrophoresis was performed at 5 V $cm^{-1}$ for 3 to 4 h, depending on the length of the amplified nucleotide. The gel was stained with ethidium bromide and visualized under UV light.

### PCR-FSSCP analysis

100 ng of genomic DNA was amplified in a total volume of 50 μl in the buffer recommended by Perkin-Elmer Cetus as described above, using the reverse primers labelled at 5′ ends with fluorescein. The PCR products were diluted 50-fold by a stop solution (Pharmacia Biotech, Uppsala, Sweden), heated at 95˚C for 5 min, and then placed on ice for 5 min. Then 4 μl of this solution was applied to each lane of the FSSCP gel fitted to an ALF II automated DNA sequencer (Pharmacia Biotech, Uppsala, Sweden). The FSSCP gels used 7% polyacrylamide gel containing 5% glycerol. Electrophoresis was performed at 30 W for 3 to 4 h, depending on the length of the amplified nucleotide. The temperature of the gel was kept at 25˚C with a built-in water jacket connected to an external thermostat-regulated water circulation system.

### Cloning and DNA sequencing of PCR products

PCR using EX-05f/EX-05r, EX-06f/EX-06r and EX-07f/EX-07r as primer pairs was performed as described above. The Amplified fragments were cloned in the TA cloning site of the pCR II™ cloning vector (Invitrogen, USA) using T4 DNA ligase (Takara Shuzo, Tokyo, Japan). Approximately 20 recombinant colonies were selected and cultured in TB (Terrific Broth, USA) medium. These recombinant clones were amplified using EX-05f/EX-F05r, EX-06f/EX-F06r or EX-07f/EX-F07r primer pairs, and PCR products were then analysed by FSSCP techniques. Sequences analysis of these PCR-amplified fragments in the cloning vector was performed using the ALF II automated DNA sequencer analyser with M13 primers.

### Statistics

Statistical analyses were performed using Student's *t*-test.

### RESULTS

### BGP and p53 protein expression in colorectal carcinoma, transitional mucosa and normal mucosa

The 17 carcinomas included 12 colonic carcinomas and 5 rectal carcinomas. The reactivity of colonic carcinoma against anti-BGP Ab and p53 is shown in Figures 1A and 1B. The positive reactivity of BGP and p53 was observed in the cytoplasm and nucleus of cancer cells, respectively. Although no immunohistochemical staining of BGP or p53 was observed in normal colonic and rectal mucosa remote from the cancer foci even in their proliferating zone, the transitional mucosa of the carcinomas in all of the 17 cases contained localized BGP foci sporadically (Figure 1A). The BGP foci adjacent to the carcinomas is shown in Figures 2A and 2B. The BGP staining pattern of the colorectal tubules in the BGP foci were observed typically in the proliferating cell zone, but sometimes it also expanded to the superficial epithelial cells. The mean diameter of the investigated BGP foci and distance from the main tumours were 2.8 ± 1.7 mm and 23.7 ± 19.6 mm (mean ± SD), respectively. The mean number of the BGP foci in each immunohistochemical section of the 17 carcinomas was 3.9 ± 2.7 (mean ± SD). No p53 protein overexpression, however, was detected in the BGP foci when using a heat antigen retrieval method (Figure 1B). Similar histologic characteristics to transitional mucosa such as crypt distortion and basal cell hyperplasia was observed in BGP foci in comparison with the BGP negative normal mucosa.

### Proliferating state in the BGP foci

The proliferative compartment in the BGP-negative normal colorectal mucosa were restricted to the lower layer (Figures 3C and 3D). In the BGP foci, on the other hand, the PCNA labelling expanded to the upper layer (Figures 3A and 3B) and the labelling index (49.3 ± 15.4%) was significantly higher than that of colorectal normal mucosa without BGP expression (34.8 ± 12.6%) ($P < 0.001$) (Figure 4). PCNA labelling index of the colorectal carcinoma was 60.1 ± 8.6%.

© 2001 Cancer Research Campaign 

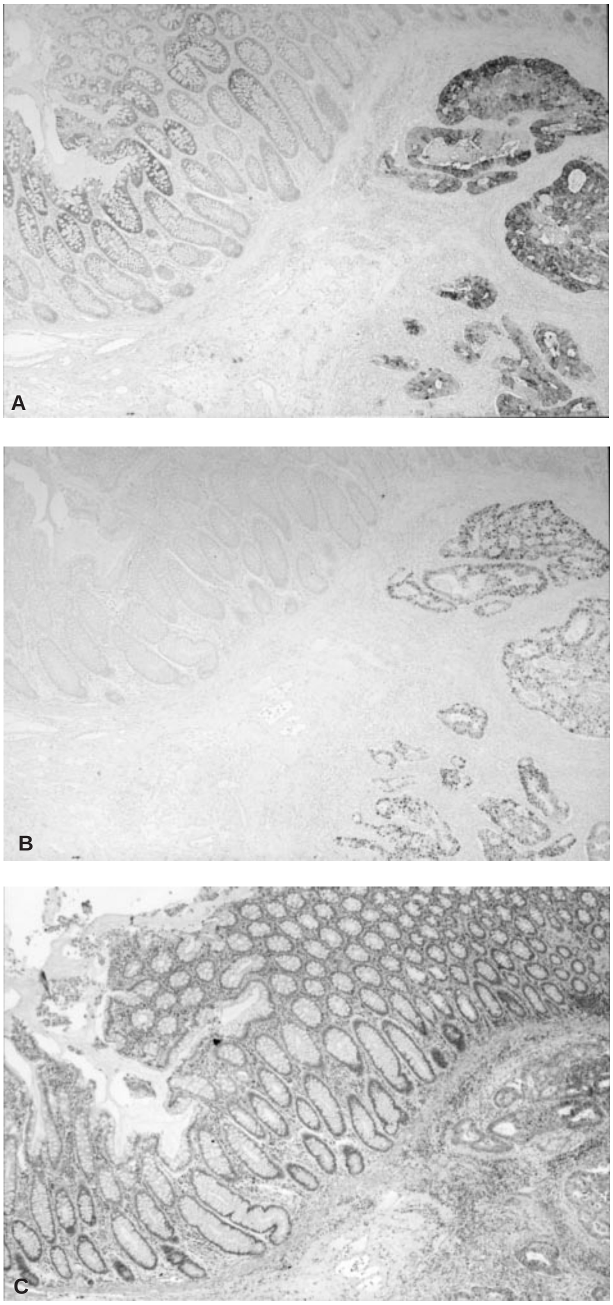

**Figure 1** Immunohistochemical staining of colonic carcinoma and the transitional mucosa with anti-brain-type glycogen phosphorylase (BGP) antibody (**A**) and anti-p53 antibody (**B**). Positive reactivity of BGP was observed in the cytoplasm of carcinoma and the transitional mucosa (**A**). p53 protein accumulation was observed in the nuclei of the carcinoma, but not in the BGP positive mucosa (**B**). (**C**) Haematoxylin-eosin staining. Magnification 15×

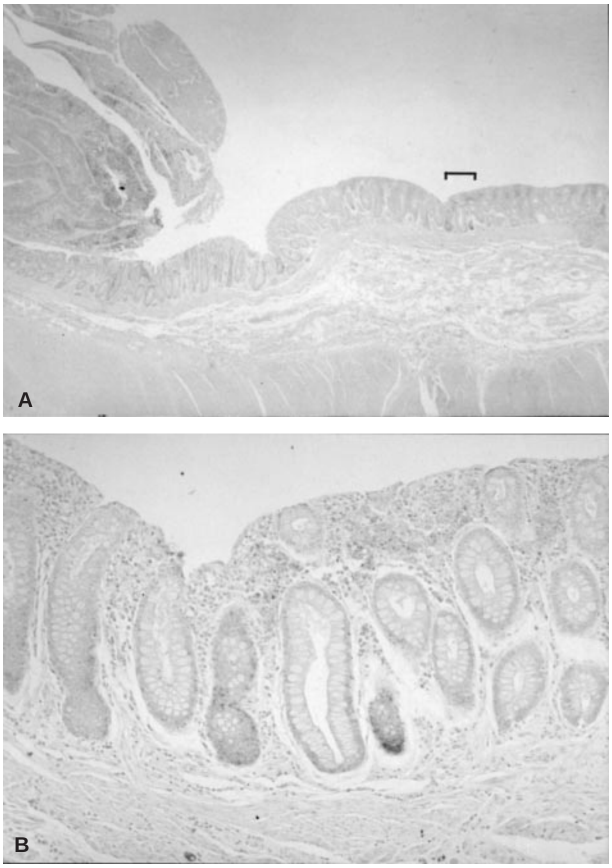

**Figure 2** Mesoscopical demonstration of a BGP stained section which contains both carcinoma and BGP positive foci (**A**) (magnification 4× ). Higher magnification of a single BGP positive focus which is marked in **A** (**B**) (magnification 35×)

### p53 and K-ras mutation in the BGP positive foci in the transitional mucosa of 'de novo' carcinoma

K-ras mutation in codon 12 was not detected in any of the specimens including carcinoma, BGP foci and BGP negative colorectal mucosa. On the other hand, p53 mutation was detected in 8 out of 17 (47.1%) in the carcinomas expressing p53 protein. Interestingly, in spite of no p53 overexpression in the BGP foci, there were frequent mutations in these foci. 7 out of 17 foci (41.2%) had the p53 point mutations. There was no mutation in the BGP negative mucosa adjacent to the carcinoma. In the carcinomas, the mutations existed in exon 5 (4 cases), exon 7 (2 cases), and exon 8 (2 cases). In the BGP foci, the mutations were detected in exon 5 (4 cases), exon 6 (1 case), exon 7 (1 case) and exon 8 (1 case) (Table 1). The mutations of p53 in carcinoma and BGP foci corresponded in three cases (cases 3, 12 and 15). Nevertheless, the mutation of one BGP focus was disconcordant with the carcinoma (case 1), and in 3 BGP foci only (cases 2, 7 and 17) and in 4 carcinomas only (cases 5, 6, 8 and 16). Because of the considerably small amount of mutated PCR products from the microdissected BGP foci in which normal cells were supposed to be contained, 4 of 7 mutated codons were not sequenced in the BGP foci. In cases 3 and 17, missence mutations in exon 5, codon 148 (Asp to Asn) and in exon 7, codon 258 (Glu to Lys) were detected, respectively. The missence and silent mutation in exon 6, codon 189 (Ala to Thr) and 187 (Gly to Gly) was sequenced in case 7.

### DISCUSSION

Concerning the carcinogenesis of sporadic colorectal carcinoma, attention has been focused on another pathway, i.e. 'de novo' carcinogenesis (Shamsuddin et al, 1985; Shimoda et al, 1989; Rembacken et al, 2000). A fair number of the colonic carcinomas are assumed to arise directly from colonic mucosa unlike ex-adenoma. If these assumptions are correct, it would be natural to

 *© 2001 Cancer Research Campaign*

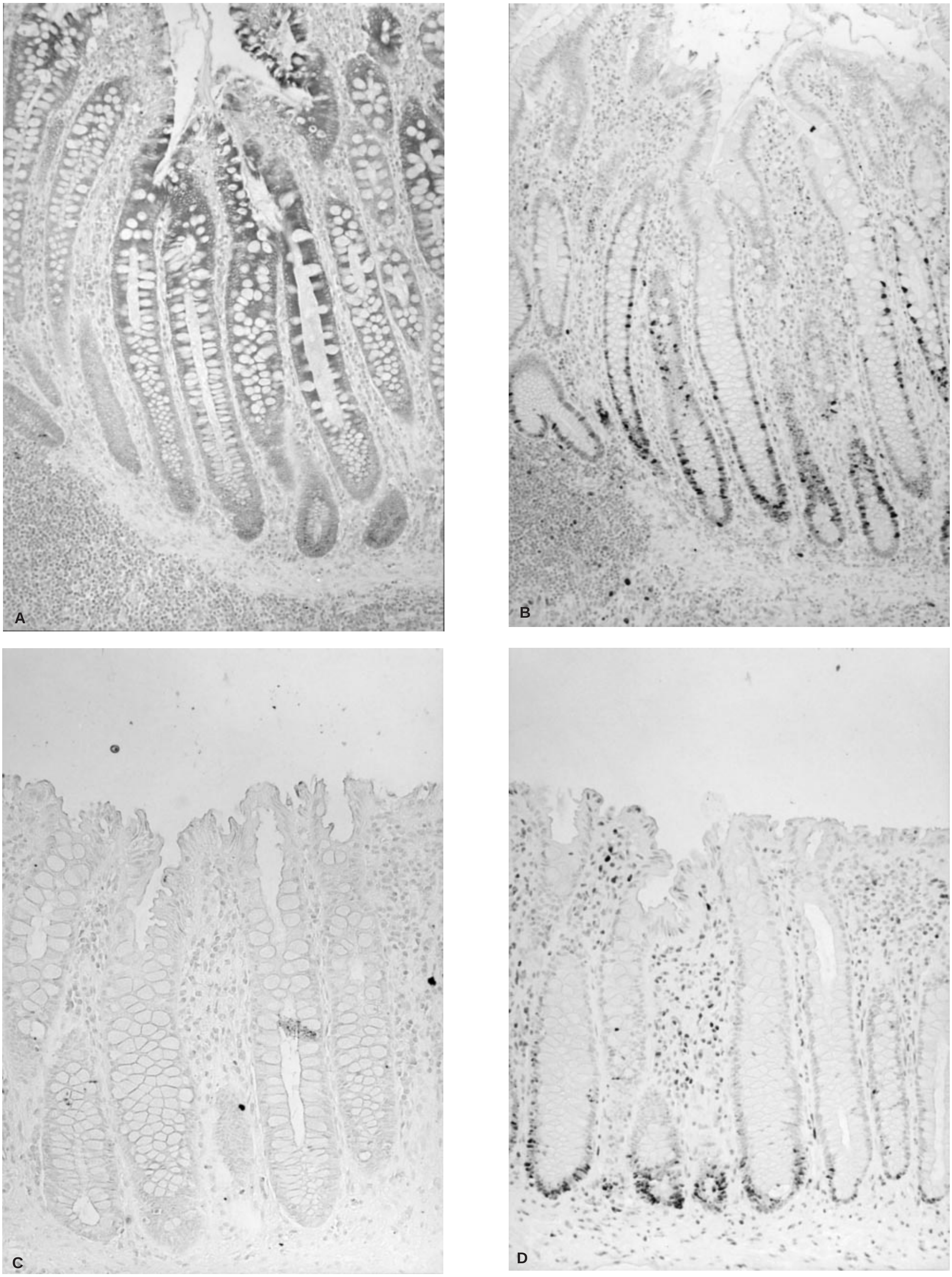

**Figure 3** Immunohistochemical analyses of colonic mucosa adjacent to carcinoma with anti-BGP antibody (**A** and **C**) and anti-PCNA Ab (**B** and **D**). The positive BGP staining was observed in these colonic tubules (**A**) (BGP focus). The PCNA labelling expanded to the upper layer in these colonic tubules of BGP focus. (Magnification 50×)

© 2001 Cancer Research Campaign 

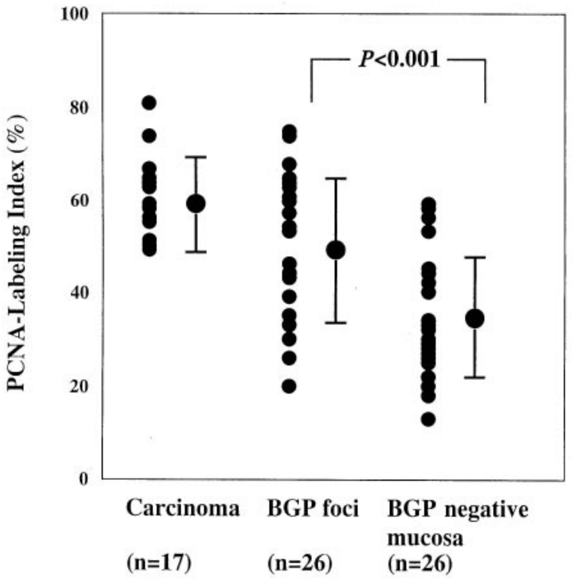

**Figure 4** Distribution of PCNA labelling index in colorectal carcinoma and adjacent mucosa with or without BGP expression consider that there are considerable reserve foci undergoing genetic alterations towards malignant transformation in the macroscopically normal-looking mucosa bearing a carcinoma, since no dysplastic change different from adenoma in the ACS has been reported yet. In the present study, multifocal expression of the BGP was observed in the colorectal mucosa adjacent to 'de novo' carcinoma. The BGP foci may be involved in the 'transitional mucosa' (Filipe and Cooke, 1974) as mentioned earlier, because the similar histologic characteristics to transitional mucosa such as crypt distortion and basal cell hyperplasia were observed in the BGP foci. The sites of BGP expression in tubules usually localized in the generative zone, where a carcinoma is supposed to arise from (Ming et al, 1967; Nagayo, 1975; Oohara et al, 1982; Shimada et al, 1984, 1992; Matsuzaki et al, 1998). The expression of BGP during ACS has recently shown excellent correlation with the increased atypism and was found prior to p53 expression (Tashima et al, 2000). These findings indicate that the expression of BGP may be the phenotypic abnormality that precedes definite morphological changes of neoplasm. It is therefore important to elucidate the genetic changes occurring in these interesting foci.

It has been suggested that genetic alterations of 'de novo' colorectal carcinoma is involved in infrequent K-ras mutation in the carcinogenesis (Minamoto et al, 1994; Yamagata et al, 1994). Aberrant crypt foci have been advocated as one putative preneoplastic lesion of the 'de novo' colorectal carcinoma without macroscopic morphological changes (Bird, 1987; Pretlow et al, 1991, 1993; Shivapurkar et al, 1997). However, these foci may not be related *to* the carcinogenesis of 'de novo' carcinoma, since those include frequent K-ras mutation in codon 12 (Pretlow et al, 1993). The present study clearly showed that there was no codon 12 of K-ras mutation in the carcinomas without any adenoma component, otherwise frequent *p53* mutation was observed in the carcinomas. We investigated only the codon 12 of K-ras, since it has been described that no mutation in the codon 13 is detected in addition to infrequent mutation of codon 12 in the 'de novo' carcinoma (Minamoto et al, 1994; Yamagata et al, 1994). Surprisingly, although none of the overexpressions of *p53* protein was observed in the BGP positive foci, the *p53* gene frequently (41.2%) mutated despite no K-ras mutation in codon 12. Since the sensitivity of SSCP is limited and influenced by the experimental condition, the authors estimated the detectability of these mutations as about 80% based on the previous report (Jordanova et al, 1997).

There have been many reports discussing the correlation between p53 protein overexpression and the p53 mutation. Although it is generally accepted that the protein and mutation correspond in carcinomas, some have described the incorrespondence between the two. For example, Dunn et al (1994) examined the correlation between p53 mutation and antibody staining using 81 primary breast carcinomas, and p53 mutations were associated with positive p53 protein staining in only 2 patients. Other researchers also concluded that accumulation of p53 protein, as detectable by immunohistochemistry, is not a reliable indicator for *p53* gene mutation in human breast cancer (Lohmann et al, 1993; Lisboa et al, 1997). In the present study, only about half on the p53 protein-positive colorectal carcinomas showed p53 mutation. Concerning precancerous lesions in the gastrointestinal tract, it has been described that p53 mutation in the intestinal metaplasia, which is regarded as a major precursor of intestinal-type gastric carcinoma, has been considerably frequently observed, but the overexpression of p53 protein is rarely detected in it (Shiao et al, 1994).

Proliferative characterization of BGP foci was conducted using PCNA labelling index and was compared with that of BGP-negative mucosa. PCNA staining revealed that there was an expansion of proliferative compartment in BGP foci and that these were significantly higher in a proliferating state than colorectal mucosa without BGP. The present study detected the p53 mutation frequently in the BGP foci including generative cell zone, as mentioned above. Both the markers of PCNA and p53 have been considered to be crucial to the demonstration of cell proliferation in the cell cycle phases (Baker et al, 1990; Fearon and Vogelstein, 1990; Hollstein et al, 1991; Brito et al, 1992; Greenblatt et al, 1994; Levine et al, 1994). These observations suggest that the generative cells in the BGP foci may deviate from the differentiation and be blocked from apoptotic cell death. Thus, these novel foci with BGP probably contribute to the research of the carcinogenesis on the 'de novo' colorectal carcinogenesis.

Studies on very early preneoplastic lesions are essential for understanding the exact details of the molecular mechanism of colorectal carcinogenesis. BGP-positive foci might be such a candidate, being potentially early preneoplastic lesions with no apparent neoplastic morphological change. Since 'de novo' carcinoma is thought to arise directly from normal colorectal mucosa shortly after p53 and APC mutation, or p53 mutation alone (Aoki et al, 1994; Umetani et al, 2000), further investigations are necessary to determine the relationship between p53 and APC mutation in the BGP foci.

### ACKNOWLEDGEMENTS

This work was supported in part by Grants-in-Aid (No. 11671254 and 12877194) for Scientific Research from the Ministry of Education, Japan.

 *© 2001 Cancer Research Campaign*

**Table 1** *p53* and *K-ras* gene mutation in BGP positive foci

| Case no. | Location | Histological grading TMN/grade/Dukes' | Immunoreactivity | | *p53* gene | *K-ras* gene |
|---|---|---|---|---|---|---|
| | | | BGP | p53 | exon/codon/mutation | |
| 1. T | A | T1N0M0/I/A | + | + | 8/ 282/ CGG(Arg) to TGG(Trp) missense | wild |
| N(BGP+) | | | + | – | 5/NA | wild |
| N(BGP–) | | | – | – | wild | wild |
| 2. T | C | T1N0M0/I/A | + | + | wild | wild |
| N(BGP+) | | | + | – | 5/NA | wild |
| N(BGP–) | | | – | – | wild | wild |
| 3. T | D | T2N0M0/I/A | + | + | 7/ 258/ GAA(Glu) to AAA(Lys) missense | wild |
| N(BGP+) | | | + | – | 7/ 258/ GAA(Glu) to AAA(Lys) missense | wild |
| N(BGP–) | | | – | – | wild | wild |
| 4.T | S | T1N2M0/III/C | + | + | wild | wild |
| N(BGP+) | | | + | – | wild | wild |
| N(BGP–) | | | – | – | wild | wild |
| 5.T | A | T2N0M0/I/A | + | + | 5/NA | wild |
| N(BGP+) | | | + | + | wild | wild |
| N(BGP–) | | | – | – | wild | wild |
| 6.T | S | T1N0M0/I/A | + | + | 7/238/ TGT(Cys) to TAT(Tyr) missense | wild |
| N(BGP+) | | | + | – | wild | wild |
| N(BGP–) | | | – | – | wild | wild |
| 7.T | R | T1N0M0/I/A | + | + | wild | wild |
| N(BGP+) | | | + | – | 6/187/ GGT(Gly) to GGC(Gly) silent<br>6/189/ GCC(Ala) to ACC(Thr) missense | wild |
| N(BGP–) | | | – | – | wild | wild |
| 8.T | S | T2N1M0/III/C | + | + | 5/ 163/ TAC(Tyr) to TGC(Cys) missense | wild |
| N(BGP+) | | | + | – | wild | wild |
| N(BGP–) | | | – | – | wild | wild |
| 9.T | R | T1N0M0/I/A | + | + | wild | wild |
| N(BGP+) | | | + | – | wild | wild |
| N(BGP–) | | | – | – | wild | wild |
| 10.T | A | T1N0M0/I/A | + | + | wild | wild |
| N(BGP+) | | | + | – | wild | wild |
| N(BGP–) | | | – | – | wild | wild |
| 11.T | T | T1N0M0/I/A | + | + | wild | wild |
| N(BGP+) | | | + | – | wild | wild |
| N(BGP–) | | | – | – | wild | wild |
| 12.T | A | T1N1M0/III/C | + | + | 8/ 265/ CTACTGGGA(Leu Leu Gly)<br>to CTACACTGGA 2bp insertion | wild |
| N(BGP+) | | | + | – | 8/ NA | wild |
| N(BGP–) | | | – | – | wild | wild |
| 13.T | S | T1N0M0/I/A | + | + | wild | wild |
| N(BGP+) | | | + | – | wild | wild |
| N(BGP–) | | | – | – | wild | wild |
| 14.T | D | T1N0M0/I/A | + | + | wild | wild |
| N(BGP+) | | | + | – | wild | wild |
| N(BGP–) | | | – | – | wild | wild |
| 15.T | S | T2N0M0/II/B | + | + | 5/ NA | wild |
| N(BGP+) | | | + | – | 5/ NA | wild |
| N(BGP–) | | | – | – | wild | wild |
| 16.T | R | T1N0M0/I/A | + | + | 5/ NA | wild |
| N(BGP+) | | | + | – | wild | wild |
| N(BGP–) | | | – | – | wild | wild |
| 17.T | S | T1N0M0/I/A | + | + | wild | wild |
| N(BGP+) | | | + | – | 5/ 148/ GAT(Asp) to AAT(Asn) missense | wild |
| N(BGP–) | | | – | – | wild | wild |

NA = not available, T = tumour, N = normal mucosa, BGP = brain (fetal)-type glycogen phosphorylase, C = caecum, A = ascending colon, Tr = transverse colon, D = descending colon, S = sigmoid colon, R = rectum.

### REFERENCES

Aoki T, Takeda S, Yanagisawa A, Kato Y, Ajioka Y, Watanabe H and Nakamura Y (1994) APC and p53 mutations in *de novo* colorectal adenocarcinomas. *Hum Mutat* **3**: 342–346

Baker SJ, Preisinger AC and Jessup M (1990) p53 gene mutations occur in combination with 17p allelic deletions as late events in colorectal tumorigenesis. *Cancer Res* **50**: 7717–7722

© 2001 Cancer Research Campaign

Bird RP (1987) Observation and quantification of aberrant crypts in the murine colon treated with a colon carcinogen. *Cancer Lett* **37**: 147–151

Brito MJ, Filipe MI and Morris RW (1992) Cell proliferation study on gastric carcinoma and non-involved gastric mucosa using a bromodeoxyuridine (BrdU) labeling technique. *Eur J Cancer Prev* **1**: 429–435

Buchman VL, Chumakov PM, Nikkina NN, Samarina PP and Georgiev GP (1988) A variation in the structure of the protein-coding of the human p53 gene. *Gene* **70**: 245–252

Cori CF and Cori GT (1936) Mechanism and formation of hexosemonophosphate in muscle and isolation of a new phosphate ester. *Proc Soc Exp Biol Med* **34**: 702–712

Davis CH, Schliselfeld LH, Wolf DP, Leavitt CA and Krebs EG (1967) Interrelationships among glycogen phosphorylase isozymes. *J Biol Chem* **242**: 4824–4833

Dunn JM, Hastrich DJ, Newcomb P, Webb JC, Maitland NJ and Farndon JR (1994) Correlation between p53 mutations and antibody staining in breast carcinoma. *Br J Surg* **81**: 1410–1412

Fearon ER and Jones PA (1992) Progressing toward a molecular description of colorectal cancer development. *FASEB J* **6**: 2783–2790

Fearon ER and Vogelstein B (1990) A genetic model for colorectal Tumorigenesis. *Cell* **61**: 759–767

Filipe MI and Cooke KB (1974) Changes in composition of mucin in the mucosa adjacent to carcinoma of the colon as compared with the normal: a biochemical investigation. *J Clin Pathol* **27**: 315–318

Gelinas RP, Froman BE, McElroy F, Tait RC and Gorin FA (1989) Human brain glycogen phosphorylase: characterization of fetal cDNA and genomic sequence. *Mol Brain Res* **6**: 177–185

Greenblatt MS, Bennett WP, Hollstein M and Harris CC (1994) Mutations in the p53 tumor suppressor gene: clues to cancer etiology and molecular pathogenesis. *Cancer Res* **54**: 4855–4878

Hollstein M, Sidransky D, Vogelstein B and Harris CC (1991) p53 mutations in human cancers. *Science* **253**: 49–53

Ignacio PC, Baldwin BA, Vijayan VK, Tait RC and Gorin FA (1990) Brain isozyme of glycogen phosphorylase: immunohistochemical localization within the central nervous system. *Brain Res* **529**: 42–49

Jordanova A, Kalaydjieva L, Savov A, Claustres M, Schwarz M, Estivill X, Angelicheva D, Haworth A, Casals T and Kremensky I (1997) SSCP analysis: a blind sensitivity trial. *Human Mutat* **10**: 65–70

Krebs EG and Fisher EH (1956) The phosphorylase b to be a converting enzyme of rabbit skeletal muscle. *Biochem Biophys Acta* **20**: 150

Kuramoto S and Oohara T (1988) Minute cancers arising de novo in the human large intestine. *Cancer* **61**: 829–834

Levine AJ, Perry ME, Chang A, Silver A, Dittmer D, Wu M and Welsh D (1994) The role of the p53 suppressor gene in tumorigenesis. The 1993 Walter Hubert Lecture. *Br J Cancer* **69**: 409–416

Lisboa BW, Vogtlander S, Gilster T, Riethdorf L, Milde-Langosch K and Loning T (1997) Molecular and immunohistochemical analysis of p53 mutations in scrapings and tissue from preinvasive and invasive breast cancer. *Virchows Archiv* **431**: 375–381

Lohmann D, Ruhri C, Schmitt M, Graeff H and Hofler H (1993) Accumulation of p53 protein as an indicator for p53 gene mutation in breast cancer. Occurrence of false-positives and false-negatives. *Diagnostic Mol Pathol* **1**: 36–41

Matsuzaki H, Shimada S, Uno K, Tsuruta J and Ogawa M (1998) Novel subtyping of intestinal metaplasia in the human stomach: brain-type glycogen phosphorylase expression in the proliferative zone and its relationship with carcinogenesis. *Am J Clin Pathol* **109**: 181–189

Minamoto T, Sawaguchi K, Mai M, Yamashita N, Sugimura T and Esumi H (1994) Infrequent K-ras activation in superficial-type(flat) colorectal adenomas and adenocarcinomas. *Cancer Res* **54**: 2841–2844

Ming SC, Goldman H and Freiman DG (1967) Intestinal metaplasia and histogenesis of carcinoma in human stomach. *Cancer* **20**: 1418–1429

Morson BC (1974) The polyp-cancer sequence in the large bowel. *Proc R Soc Med* **67**: 451–457

Mueller J, Mueller E, Hoepner I, Jutting J, Bethke B, Stolte M and Hofler H (1996) Expression of bcl-2 and p53 in de novo and ex-adenoma colon carcinoma: a comparative immunohistochemical study. *J Pathol* **180**: 259–265

Nagayo T (1975) Microscopical cancer of the stomach; a study on histogenesis of gastric carcinoma. *Int J Cancer* 16: 52–60

Nakano K, Hwang P, and Fletterick RJ (1986) Complete cDNA sequence for rabbit muscle glycogen phosphorylase. *FEBS Lett* **204**: 283–287

Newgard CB, Littman DR, Genderen CV, Smith M and Fletterick RJ (1988) Human brain glycogen phosphorylase. *J Biol Chem* **263**: 3850–3857

Newgard CB, Hwang PK and Fletterick RJ (1989) The family of glycogen phosphorylase: structure and function. *Crit Rev Biochem Mol Biol* **24**: 69–99

Oohara T, Tohma H, Takezoe K, Ukawa S, Johjima Y, Asakura R, Ano G and Kurosawa H (1982) Minute gastric cancers less than 5 mm in diameter. *Cancer* **50**: 801–810

Pretlow TP, Barrow BJ, Ashton WS, O'Riordan MA, Pretlow TG, Jurcisek JA and Stellato TA (1991) Aberrant crypts: putative preneoplastic foci in human colonic mucosa. *Cancer Res* **51**: 564–567

Pretlow TP, Brasitus TA, Fulton NC, Cheyer C and Kaplan EL (1993) K-ras mutations in putative preneoplastic lesions in human colon: *J Natl Cancer Inst* **85**:2004–2007

Rembacken BJ, Fujii T, Cairn A, Dixon MF, Yoshida S, Chalmers DM and Axon ART (2000) Flat and depressed colonic neoplasms: prospective study of 1000 colonoscopies in the UK. *Lancet* **355**: 1211–1214

Sambrook J, Fitsch EF and Maniatis T (1989) Molecular cloning. In a laboratory Manual. Cold Spring Harbor Laboratory Press, New York.

Sato K, Morris HP and Weinhouse S (1972) Phosphorylase: a new isozyme in rat hepatic tumors and fetal liver. *Science* **178**: 879–881

Shamsuddin AM, Kato Y, Kunishima N, Sugano H and Trump B (1985) Carcinoma in situ in non-polypoid mucosa of the large intestine. *Cancer* **56**: 2849–2854

Shi SR, Key ME and Kalra KL (1991) Antigen retrieval in formalin-fixed, paraffin-embedded tissues. An enhancement method for immunohistochemical staining based on microwave oven heating of tissue sections. *J Histochem Cytochem* **39**: 741–748

Shiao YH, Rugge M, Correa P, Lehmann HP and Scheer WD (1994) p53 alteration in gastric precancerous lesions. *Am J Pathol* **144**: 511–517

Shimada S, Maeno M, Misumi A and Akagi M (1984) Histochemical study of phosphorylase in proliferating cells of intestinal metaplasia and carcinoma of the human stomach. *Scand J Gastroenterol* **19**: 965–970

Shimada S, Maeno M, Akagi M, Hatayama I, Sato T and Sato K (1986) Immunohistochemical detection of glycogen phosphorylase isoenzymes in rat and human tissues. *Histochem J* **18**: 334–338

Shimada S, Maeno M, Misumi A, Takano S and Akagi M (1987) Antigen reversion of glycogen phosphorylase isoenzyme in carcinoma and proliferative zone of intestinal metaplasia of the human stomach. *Gastroenterology* **93**: 35–40

Shimada S, Honmyo U, Yagi Y, Ikeda T, Yokota T and Ogawa M (1992) Expression of glycogen phosphorylase activity in minute gastric carcinoma. *Am J Gastroenterol* **87**: 1230–1231

Shimada S, Tashima S, Yamaguchi K, Matsuzaki H and Ogawa M (1999) Carcinogenesis of intestinal-type gastric cancer and colorectal cancer is commonly accompanied by expression of brain (fetal)-type glycogenphosphorylase. *J Exp Clin Cancer Res* **18**: 111–118

Shimoda T, Ikegami M, Fujisaki J, Matsui T, Aizawa S and Ishikawa E (1989) Early colorectal carcinoma with special reference to its development de novo. *Cancer* **64**: 1138–1146

Shivapurkar N, Huang L, Ruggeri B, Swalsky PA, Bakker A, Finkelstein S, Frost A and Silverberg (1997) K-ras and p53 mutations in aberrant crypt foci and colonic tumors from colon cancer patients. *Cancer Lett* **115**: 39–46

Tashima S, Shimada S, Matsuzaki H, Yamaguchi K, Tsuruta J and Ogawa M (2000) Expression of brain-type glycogen phosphorylase is a potentially novel early biomarker in the carcinogenesis of human colorectal carcinomas. *Am J Gastroenterol* **95**: 255–263

Umetani N, Sasaki S, Watanabe T, Matsuda K and Muto T (2000) Involvement of APC and K-ras mutation in non-polypoid colorectal tumorigenesis. *Br J Cancer* **82**: 9–15

Wang O, Gao H, Chen Y, Wang Y, He J and Jin C (1992) Biopathologic characteristics of DNA content in crypt cells of transitional mucosa adjacent to carcinomas of the rectum and rectosigmoid. *Dis Colon Rectum* **35**: 670–675

Vogelstein B, Fearon ER, Hamilton SR, Kern SE, Preisinger AC, Leppert M, Nakamura Y, White R, Smits AM and Bos JL (1988) Genetic alterations during colorectal-tumor development. *N Eng J Med* **319**: 525–532

Yamagata S, Muto T, Uchida Y, Masaki T, Sawada T, Tsuno N and Hirooka T (1994) Lower incidence of K-ras codon 12 mutation in flat colorectal adenomas than in polypoid adenomas. *Jpn J Cancer Res* **85**: 147–151

Zhang H, Nordenskjold B, Dufmats M, Soderkvist P and Sun XF (1998) K-ras mutations in colorectal adenocarcinomas and neighbouring transitional mucosa. *Eur J Cancer* **34**: 2053–2057

 *© 2001 Cancer Research Campaign*

## References

[PDF_00474] Aoki T., Takeda S., Yanagisawa A., Kato Y., Ajioka Y., Watanabe H., Kudo S., Nakamura Y. (1994). APC and p53 mutations in de novo colorectal adenocarcinomas.. Hum Mutat.

[PDF_00477] Baker S. J., Preisinger A. C., Jessup J. M., Paraskeva C., Markowitz S., Willson J. K., Hamilton S., Vogelstein B. (1990). p53 gene mutations occur in combination with 17p allelic deletions as late events in colorectal tumorigenesis.. Cancer Res.

[PDF_00542] Bird R. P. (1987). Observation and quantification of aberrant crypts in the murine colon treated with a colon carcinogen: preliminary findings.. Cancer Lett.

[PDF_00544] Brito M. J., Filipe M. I., Morris R. W. (1992). Cell proliferation study on gastric carcinoma and non-involved gastric mucosa using a bromodeoxyuridine (BrdU) labelling technique.. Eur J Cancer Prev.

[PDF_00548] Buchman V. L., Chumakov P. M., Ninkina N. N., Samarina O. P., Georgiev G. P. (1988). A variation in the structure of the protein-coding region of the human p53 gene.. Gene.

[PDF_00554] Davis C. H., Schliselfeld L. H., Wolf D. P., Leavitt C. A., Krebs E. G. (1967). Interrelationships among glycogen phosphorylase isozymes.. J Biol Chem.

[PDF_00560] Fearon E. R., Jones P. A. (1992). Progressing toward a molecular description of colorectal cancer development.. FASEB J.

[PDF_00562] Fearon E. R., Vogelstein B. (1990). A genetic model for colorectal tumorigenesis.. Cell.

[PDF_00564] Filipe M. I., Cooke K. B. (1974). Changes in composition of mucin in the mucosa adjacent to carcinoma of the colon as compared with the normal: a biochemical investigation.. J Clin Pathol.

[PDF_00567] Gelinas R. P., Froman B. E., McElroy F., Tait R. C., Gorin F. A. (1989). Human brain glycogen phosphorylase: characterization of fetal cDNA and genomic sequences.. Brain Res Mol Brain Res.

[PDF_00570] Greenblatt M. S., Bennett W. P., Hollstein M., Harris C. C. (1994). Mutations in the p53 tumor suppressor gene: clues to cancer etiology and molecular pathogenesis.. Cancer Res.

[PDF_00573] Hollstein M., Sidransky D., Vogelstein B., Harris C. C. (1991). p53 mutations in human cancers.. Science.

[PDF_00575] Ignacio P. C., Baldwin B. A., Vijayan V. K., Tait R. C., Gorin F. A. (1990). Brain isozyme of glycogen phosphorylase: immunohistological localization within the central nervous system.. Brain Res.

[PDF_00578] Jordanova A., Kalaydjieva L., Savov A., Claustres M., Schwarz M., Estivill X., Angelicheva D., Haworth A., Casals T., Kremensky I. (1997). SSCP analysis: a blind sensitivity trial.. Hum Mutat.

[PDF_00581] KREBS E. G., FISCHER E. H. (1956). The phosphorylase b to a converting enzyme of rabbit skeletal muscle.. Biochim Biophys Acta.

[PDF_00583] Kuramoto S., Oohara T. (1988). Minute cancers arising de novo in the human large intestine.. Cancer.

[PDF_00585] Levine A. J., Perry M. E., Chang A., Silver A., Dittmer D., Wu M., Welsh D. (1994). The 1993 Walter Hubert Lecture: the role of the p53 tumour-suppressor gene in tumorigenesis.. Br J Cancer.

[PDF_00588] Lisboa B. W., Vogtländer S., Gilster T., Riethdorf L., Milde-Langosch K., Löning T. (1997). Molecular and immunohistochemical analysis of p53 mutations in scrapings and tissue from preinvasive and invasive breast cancer.. Virchows Arch.

[PDF_00592] Lohmann D., Ruhri C., Schmitt M., Graeff H., Höfler H. (1993). Accumulation of p53 protein as an indicator for p53 gene mutation in breast cancer. Occurrence of false-positives and false-negatives.. Diagn Mol Pathol.

[PDF_00596] Matsuzaki H., Shimada S., Uno K., Tsuruta J., Ogawa M. (1998). Novel subtyping of intestinal metaplasia in the human stomach: brain-type glycogen phosphorylase expression in the proliferative zone and its relationship with carcinogenesis.. Am J Clin Pathol.

[PDF_00600] Minamoto T., Sawaguchi K., Mai M., Yamashita N., Sugimura T., Esumi H. (1994). Infrequent K-ras activation in superficial-type (flat) colorectal adenomas and adenocarcinomas.. Cancer Res.

[PDF_00603] Ming S. C., Goldman H., Freiman D. G. (1967). Intestinal metaplasia and histogenesis of carcinoma in human stomach. Light and electron microscopic study.. Cancer.

[PDF_00605] Morson B. (1974). President's address. The polyp-cancer sequence in the large bowel.. Proc R Soc Med.

[PDF_00607] Mueller J., Mueller E., Hoepner I., Jütting J., Bethke B., Stolte M., Höfler H. (1996). Expression of bcl-2 and p53 in de novo and ex-adenoma colon carcinoma: a comparative immunohistochemical study.. J Pathol.

[PDF_00610] Nagayo T. (1975). Microscopical cancer of the stomach--a study on histogenesis of gastric carcinoma.. Int J Cancer.

[PDF_00612] Nakano K., Hwang P. K., Fletterick R. J. (1986). Complete cDNA sequence for rabbit muscle glycogen phosphorylase.. FEBS Lett.

[PDF_00616] Newgard C. B., Hwang P. K., Fletterick R. J. (1989). The family of glycogen phosphorylases: structure and function.. Crit Rev Biochem Mol Biol.

[PDF_00614] Newgard C. B., Littman D. R., van Genderen C., Smith M., Fletterick R. J. (1988). Human brain glycogen phosphorylase. Cloning, sequence analysis, chromosomal mapping, tissue expression, and comparison with the human liver and muscle isozymes.. J Biol Chem.

[PDF_00618] Oohara T., Tohma H., Takezoe K., Ukawa S., Johjima Y., Asakura R., Aono G., Kurosaka H. (1982). Minute gastric cancers less than 5 mm in diameter.. Cancer.

[PDF_00623] Pretlow T. P., Brasitus T. A., Fulton N. C., Cheyer C., Kaplan E. L. (1993). K-ras mutations in putative preneoplastic lesions in human colon.. J Natl Cancer Inst.

[PDF_00626] Rembacken B. J., Fujii T., Cairns A., Dixon M. F., Yoshida S., Chalmers D. M., Axon A. T. (2000). Flat and depressed colonic neoplasms: a prospective study of 1000 colonoscopies in the UK.. Lancet.

[PDF_00631] Sato K., Morris H. P., Weinhouse S. (1972). Phosphorylase: a new isozyme in rat hepatic tumors and fetal liver.. Science.

[PDF_00633] Shamsuddin A. M., Kato Y., Kunishima N., Sugano H., Trump B. F. (1985). Carcinoma in situ in nonpolypoid mucosa of the large intestine. Report of a case with significance in strategies for early detection.. Cancer.

[PDF_00635] Shi S. R., Key M. E., Kalra K. L. (1991). Antigen retrieval in formalin-fixed, paraffin-embedded tissues: an enhancement method for immunohistochemical staining based on microwave oven heating of tissue sections.. J Histochem Cytochem.

[PDF_00639] Shiao Y. H., Rugge M., Correa P., Lehmann H. P., Scheer W. D. (1994). p53 alteration in gastric precancerous lesions.. Am J Pathol.

[PDF_00650] Shimada S., Honmyo U., Yagi Y., Ikeda T., Ogawa M., Yokota T. (1992). Expression of glycogen phosphorylase activity in minute gastric carcinoma.. Am J Gastroenterol.

[PDF_00644] Shimada S., Maeno M., Akagi M., Hatayama I., Sato T., Sato K. (1986). Immunohistochemical detection of glycogen phosphorylase isoenzymes in rat and human tissues.. Histochem J.

[PDF_00641] Shimada S., Maeno M., Misumi A., Akagi M. (1984). Histochemical study of phosphorylase in proliferating cells of intestinal metaplasia and carcinoma of the human stomach.. Scand J Gastroenterol.

[PDF_00647] Shimada S., Maeno M., Misumi A., Takano S., Akagi M. (1987). Antigen reversion of glycogen phosphorylase isoenzyme in carcinoma and proliferative zone of intestinal metaplasia of the human stomach. An immunohistochemical study.. Gastroenterology.

[PDF_00653] Shimada S., Tashima S., Yamaguchi K., Matsuzaki H., Ogawa M. (1999). Carcinogenesis of intestinal-type gastric cancer and colorectal cancer is commonly accompanied by expression of brain (fetal)-type glycogen phosphorylase.. J Exp Clin Cancer Res.

[PDF_00657] Shimoda T., Ikegami M., Fujisaki J., Matsui T., Aizawa S., Ishikawa E. (1989). Early colorectal carcinoma with special reference to its development de novo.. Cancer.

[PDF_00660] Shivapurkar N., Huang L., Ruggeri B., Swalsky P. A., Bakker A., Finkelstein S., Frost A., Silverberg S. (1997). K-ras and p53 mutations in aberrant crypt foci and colonic tumors from colon cancer patients.. Cancer Lett.

[PDF_00663] Tashima S., Shimada S., Yamaguchi K., Tsuruta J., Ogawa M. (2000). Expression of brain-type glycogen phosphorylase is a potentially novel early biomarker in the carcinogenesis of human colorectal carcinomas.. Am J Gastroenterol.

[PDF_00667] Umetani N., Sasaki S., Masaki T., Watanabe T., Matsuda K., Muto T. (2000). Involvement of APC and K-ras mutation in non-polypoid colorectal tumorigenesis.. Br J Cancer.

[PDF_00673] Vogelstein B., Fearon E. R., Hamilton S. R., Kern S. E., Preisinger A. C., Leppert M., Nakamura Y., White R., Smits A. M., Bos J. L. (1988). Genetic alterations during colorectal-tumor development.. N Engl J Med.

[PDF_00670] Wang Q., Gao H., Chen Y., Wang Y., He J., Jin C. (1992). Biopathologic characteristics of DNA content in crypt cells of transitional mucosa adjacent to carcinomas of the rectum and rectosigmoid.. Dis Colon Rectum.

[PDF_00676] Yamagata S., Muto T., Uchida Y., Masaki T., Sawada T., Tsuno N., Hirooka T. (1994). Lower incidence of K-ras codon 12 mutation in flat colorectal adenomas than in polypoid adenomas.. Jpn J Cancer Res.

[PDF_00679] Zhang H., Nordenskjöld B., Dufmats M., Söderkvist P., Sun X. F. (1998). K-ras mutations in colorectal adenocarcinomas and neighbouring transitional mucosa.. Eur J Cancer.

